# Household air pollution is associated with disease severity in Ugandan children hospitalized with hypoxemic pneumonia

**DOI:** 10.1371/journal.pone.0348277

**Published:** 2026-05-12

**Authors:** Ayla Ahmed, Sehaj Sandhu, Sophie Namasopo, Juliet Nabwire, Qaasim Mian, Andrea L. Conroy, Jackson Amone, Charles Olaro, Robert O. Opoka, Michael T. Hawkes

**Affiliations:** 1 Department of Medicine, University of St Andrews, St Andrews, United Kingdom; 2 Department of Pediatrics, University of Alberta, Edmonton, Alberta, Canada; 3 Department of Pediatrics, Kabale District Hospital, Kabale, Uganda; 4 Global Health Uganda, Kampala, Uganda; 5 Department of Pediatrics, Indiana University School of Medicine, Indianapolis, Indiana, United States of America; 6 Ministry of Health, Kampala, Uganda; 7 Department of Pediatrics, Medical College East Africa, Aga Khan University, Nairobi, Kenya; 8 Department of Pediatrics, University of British Columbia, Vancouver, Canada; Tribhuvan University, NEPAL

## Abstract

**Background:**

Household air pollution (HAP) due to biomass fuel use is a risk factor for childhood pneumonia, a leading cause of under-five mortality globally. This study examined the relationship between HAP and disease severity in Ugandan children hospitalized with hypoxemic pneumonia.

**Methods:**

We conducted a retrospective case-control study across 20 Ugandan hospitals. PM_2.5_ exposure was estimated using caregiver-reported fuel use, cooking duration, kitchen structure, and ventilation. Pneumonia severity was assessed clinically, and associations were analyzed using non-parametric tests.

**Results:**

We included 735 children (median age 9 months, 42% female) hospitalized with hypoxemic pneumonia. Most households used firewood (84%) or charcoal (16%) for cooking. Other HAP sources included cigarette smoke (17%) and open-flame lighting (17%). The median estimated personal PM_2.5_ exposure was 145 µg/m³ (IQR 79–270), and 732 children (99.6%) exceeded the recommended WHO limit of 15 µg/m³. Chronic HAP-related symptoms included cough (57%), red eyes (41%), rhinorrhea (38%), difficulty breathing (22%), and wheeze (6.5%). Higher PM2.5 exposure was significantly associated with more frequent red eyes (p = 0.0073), rhinorrhea (p = 0.016), and difficulty breathing (p < 0.0001). The median SICK score was 3.9 (IQR 3.4–5.0). Higher scores correlated with higher PM_2.5_ exposure (τ = 0.15, p < 0.0001) and increased mortality risk (p = 0.0024). Higher PM_2.5_ exposure was also linked to WHO danger signs, lower SpO_2_ at admission, longer duration of oxygen therapy, and greater total oxygen volume administered over the hospital admission (p < 0.05 for all).

**Conclusion:**

Household PM_2.5_ exposure from biomass combustion is associated with greater pneumonia severity in hospitalized children under five. Reducing household air pollution through cleaner fuels, better ventilation, and behavioral changes may improve outcomes for a leading cause of child mortality globally.

## Introduction

Beyond the neonatal period, the leading cause of mortality in children under five years of age is pneumonia, accounting for 13% of deaths world-wide [1]. Fifteen low-income countries account for three quarters of all the worldwide pneumonia episodes among children under 5, with the highest number of cases occurring in South Asia and sub-Saharan Africa [2].

Biomass combustion is the primary source of domiciliary energy for over 2 billion people worldwide. Approximately 75% of households in low- and middle-income countries depend on this form of fuel for cooking, heating, and lighting [3,4]. Biomass can include sources such as wood, coal, and cow dung. Burning of biomass fuels releases particulate matter (PM) and toxic pollutants such as sulphur dioxide, carbon monoxide (CO), and other organic compounds into the household environment [5]. PM may be quantified as the density of fine particles <2.5 µm size (PM_2.5_) [6], which may have adverse health effects such as acute respiratory tract infection, increased airway hyperresponsiveness, and decreased lung growth [3,7]. By increasing the risk and severity of respiratory illness, household air pollution (HAP) accounts for over 4 million deaths annually [7].

In this study, we describe cooking practices, household biofuel use, and a summary estimate of HAP in a large, well characterized country-wide cohort of Ugandan children hospitalized with hypoxemic pneumonia. The primary objective was to examine the association between indoor pollution and disease severity. Findings from this study will inform public health interventions in the rural African context to address HAP as a modifiable risk factor for childhood pneumonia.

## Methods

### Study design

This was a retrospective case-control study examining the association between exposure to HAP and disease severity among children hospitalized with hypoxemic pneumonia. The study was a secondary analysis of the Solar Oxygen Study [8].

### Study participants and setting

Inclusion criteria for the parent trial (n = 2405) were: under five years of age; cough or difficulty breathing; peripheral O_2_ saturation (SpO_2_) <92%; and requiring hospitalization. Exclusion criteria were: known cyanotic congenital heart disease; or hypoxemic ischemic encephalopathy. For this secondary analysis, we further excluded patients who tested positive for malaria, patients for whom oxygen was not available, patients with SpO_2_ ≥ 90%, and those who did not complete a questionnaire for HAP exposure.

Patients included in the parent study were hospitalized at one of the following facilities in Uganda: Gombe District Hospital (DH); Karisizo DH; Kayunga DH; Ssembabule Health Centre (HC) IV; Bugobero HC IV; Bukedea HC IV; Bumanya HC IV; Kidera HC IV; Muyembe HC IV; Kamuli DH; Adumi HC IV; Atiak HC IV; Lalogi HC IV; Kitgum DH; Apac DH; Bundibugyo DH; Kagadi DH; Kitagata DH; Kyenjojo DH; Lyantonde DH.

### Measurement of exposure to HAP

We estimated the personal exposure of a young child to HAP using a log-linear model linking the kitchen concentration of PM_2.5_ to household variables (S1 Formula) [9]. This equation has been previously used for global burden of disease modeling [10], with correlation (r = 0.56) between predicted and measured values [9]. The model uses the following inputs to estimate the kitchen area PM_2.5_: fuel type, kitchen type, kitchen ventilation, and cooking duration. An additional variable from the original study, representing the state in India, was not included in our calculation. The ratio between the daily average personal exposure and kitchen concentration (0.628 for young children) was applied to estimate exposures for children in our study [10]. Household variables were collected using a standardized questionnaire, administered to the caregiver accompanying the hospitalized child, in the language best understood.

### Measurement of outcome: pneumonia severity

As the primary measure of clinical severity, we used the Signs of Inflammation in Children that Kill (SICK) score, a composite index of disease severity, developed for use in resource-constrained settings. Higher SICK scores are associated with progressively increasing risk of mortality [11]. The SICK score was calculated upon initial hospital admission as previously described [12]. The score (range 0–8.6) was computed as the weighted sum of the following clinical variables: age < 1 month (+2.2) or < 12 months (+1.0) or < 5 years (+0.3); temperature > 38°C or < 36°C (+1.2); heart rate > 160 minutes^−1^ for infants or > 150 minutes^−1^ for children (+0.2); respiratory rate > 60 minutes^−1^ for infants or > 50 minutes^−1^ for children (+0.4); systolic blood pressure < 65 mmHg for infants or < 75 mmHg for children (+1.2); oxygen saturation < 90% (+1.4); capillary refill time > 3 seconds (+1.2); and level of consciousness less than “alert” (+2.0).

### Sample size calculation

In our primary analysis, we tested the hypothesis that household air pollution (PM_2.5_, continuous variable) would be positively correlated with disease severity (SICK score, continuous variable). We estimated that we would need a sample size of 618 to detect a correlation of 0.1 or more, with 80% power at the α = 0.05 (one-sided) level of significance (package *pwrss* [13] in the R statistical environment). The formula for the sample size calculation is provided in the Supporting information (S2 Formula).

### Statistical analysis

Descriptive statistics used median and interquartile range (IQR) for continuous variables and number with percentage for binary or categorical variables. For comparative statistics, the non-parametric Mann–Whitney *U* test was used for continuous data. The two-tailed Pearson χ^2^ or Fisher exact test was used for categorical data, as appropriate. Correlations between continuous variables used the non-parametric rank correlation coefficient (Kendall’s tau-B, τ). To quantify the association between the PM_2.5_ and the SICK score, we used a linear regression model. The dependent variable was the SICK score and the independent variable was the log_10_(PM_2.5_). Logarithmic transformation was used because the PM_2.5_ was right-skewed and the measurement error scales proportionally with the magnitude of the concentration. The odds ratio (OR) and its 95% confidence interval (CI) were used to quantify the degree of association between binary variables. The OR was calculated as the cross-product of the 2 × 2 contingency table $\left(OR=\frac{ad}{bc}\right)\;$and the confidence interval was calculated using the maximum unconditional likelihood (Wald) method (normal approximation on the logarithmic scale) with the standard error calculated as ${SE}_{\text{ln}(OR)}=\sqrt{\frac{\text{1}}{a}+\frac{\text{1}}{b}+\frac{\text{1}}{c}+\frac{\text{1}}{d}}$. Non-parametric statistics were used to avoid the assumption of normally distributed data. GraphPad Prism version 6 (GraphPad Software Inc., La Jolla, CA, USA, 2012) and R (version 4.3.0) were used for data analysis and visualization.

### Ethical approval

The study was reviewed and approved by the Makerere University School of Biomedical Sciences Research Ethics Committee (SBSREC-644), Uganda National Science and Technology (HS 2569), and the University of Alberta Health Research Ethics Board (Reference Pro00084784).

Parents or legal guardians of children (all under five years of age) provided written informed consent for study participation.

The dates when data were accessed for research purposes (patient recruitment and data collection) were: 01/07/2019 to 30/11/2021.

## Results

**Fig 1:**
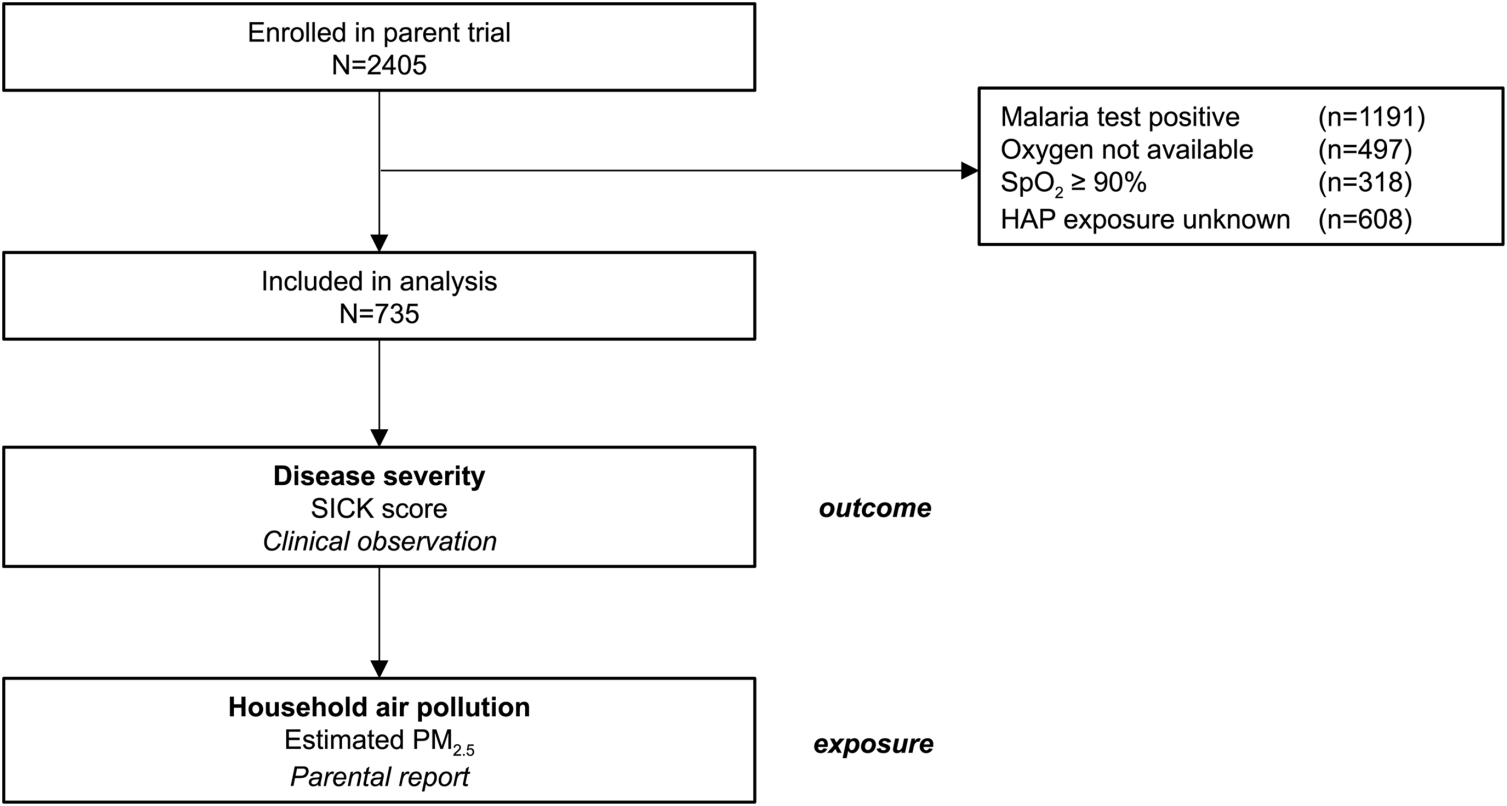
Trial flow diagram. The parent trial was the Solar Oxygen Study (N = 2405) [8], which enrolled children under 5 years of age hospitalized with oxygen saturation (SpO_2_) < 92%. Malaria-negative patients with SpO_2_ < 90% who received supplemental oxygen and who completed the questionnaire for household air pollution (HAP) were included in the current secondary analysis (N = 735). The outcome was disease severity, measured using the composite clinical score, Signs of Inflammation in Children that Kill (SICK), and assessed by the treating clinician. The exposure was household air pollution, quantified using a HAP score [9], and assessed retrospectively by parental report of household and cooking fuel characteristics.

We included 735 children hospitalized with hypoxemic pneumonia between 1 July 2019 and 30 Nov 2021 (Fig 1). Clinical characteristics were summarized (Table 1). Of note, male children were over-represented in the cohort (male to female ratio 1.4:1, p < 0.0001).

**Table 1:** Characteristics at admission of 735 hospitalized children.

	Hypoxemic pneumonia (N = 735)
***Demographics***	
Age [mo], median (IQR)	9 (3-18)
Female sex	309 (42)
Multidimensional poverty index poor ^a^	397 (54)
***Clinical features (parental report)***	
Tactile fever	606 (82)
Cough	706 (96)
Difficulty breathing	703 (96)
Convulsions	64 (8.7)
Altered consciousness	164 (22)
Vomiting everything	157 (21)
Unable to feed/drink	396 (54)
***Physical exam***	
Severely underweight ^b^	81 (11)
Oxygen saturation [%], median (IQR)	84 (79-87)
Tachypnea ^c^	439 (60)
Tachycardia ^c^	255 (35)
Temperature [°C], median (IQR)	38 (37-38)
Level of consciousness	
Alert	559 (76)
Response to voice	68 (9.3)
Response to pain	83 (11)
Unresponsive	24 (3.3)
Deep breathing	322 (44)
Chest indrawing	704 (96)
Grunting	451 (61)
Stridor	281 (38)
Cyanosis	112 (15)
WHO danger signs ^d^	605 (82)
***Composite index of disease severity***	
SICK, median (IQR)	3.9 (3.4-5.0)

Numbers represent n (%) unless otherwise stated.

IQR, interquartile range; RISC, respiratory index of severity in children; WHO, World Health Organization; SICK, Signs of Inflammation in Children that can Kill [11].

^a^The multidimensional poverty index (MPI) was calculated according to the method of Alkire and Foster [14]. A value >1/3 was considered MPI-poor.

^b^Weight-for-age below −3SD [15].

^c^Vital sign >99 percentile for age [16].

^d^WHO danger signs include convulsions, altered consciousness, vomiting everything, stridor at rest, and being unable to feed or drink.

We summarized household characteristics related to HAP (Table 2). The majority (84%) of patients used firewood as cooking biofuel. Charcoal was the second most common cooking fuel (16%). Other, cleaner, fuel sources (e.g., gas or electrical cookers) were used by less than 1% of households. The kitchen was outdoors in 33% of homes, in a structure separate from the sleeping area (main house) in 63%, and inside the main house in 3.6%. The household stove configurations were categorized as follows: three-stone fires (n = 581, 79%), traditional charcoal jikos (n = 114,16%), and fixed chimney stoves (n = 30, 4.1%). Cooking fire burned for a median of 6 hours per day (IQR 4–8), including preparation time and the time that the fire burned afterwards. Both preparation time (p = 0.016) and post-cooking burn time (p = 0.0017) were longer in households using wood compared to charcoal fuel. The number of household members partaking in daily meals was median 5 (IQR 4–7) and was correlated with preparation time (τ = 0.061, p = 0.031). The cost of the cooking fuel was associated with fuel type: 496/540 (92%) of firewood-burning households obtained the fuel for free, compared to 13/96 (14%) of charcoal-burning households (p < 0.0001). The daily cost of charcoal was median USD$0.54 (IQR 0.27–0.81).

**Table 2:** Exposure to household air pollution among 735 children hospitalized with hypoxemic pneumonia.

	Hypoxemic pneumonia (N = 735)
***Cooking practices***	
Cooking fuel	
Firewood	615 (84)
Charcoal	116 (16)
Other	4 (0.54)
Type of stove	
Stones (open fire)	581 (80)
Charcoal stove	114 (16)
Fireplace with chimney	30 (4.1)
Other	4 (0.55)
Timing of cooking	
Morning	641 (88)
Afternoon	527 (72)
Evening	476 (65)
Night	184 (25)
Cooking for how many people?	5 (4-7)
Cooking time [h], median (IQR)	4 (3-6)
Time fire burns after cooking [h], median (IQR)	0.5 (0.17-2)
Total time fire burning [h], median (IQR)	6 (4-8)
***Kitchen and ventilation***	
Kitchen location	
Separate structure	464 (63)
Outdoor kitchen	241 (33)
Indoor kitchen	26 (3.6)
Windows in bedroom	629 (86)
Additional ventilation in bedroom	606 (83)
Windows in kitchen	282 (38)
Additional ventilation in kitchen	437 (59)
***Other sources of HAP***	
Smoker	124 (17)
Lighting	
Solar powered	362 (49)
Battery	41 (5.6)
Open wick lamp	124 (17)
Hurricane lamp	83 (11)
Other	122 (17)
***Symptoms around smoke***	
Cough	239 (57)
Red eyes	182 (43)
Rhinorrhea	149 (35)
Difficulty breathing	87 (21)
Wheeze	29 (6.9)
***Composite index of exposure***	
Estimated PM_2.5_ [µg/m^3^] ^a^, median (IQR)	140 (79-270)

^a^Personal exposure to fine particulate matter (< 2.5 µm), estimated from questionnaire data.

Exposure to HAP sources other than cooking biofuels included household cigarette smoking (16%) and lighting (e.g., open wick lamp, 17%, and hurricane lamp, 11%). A large number of households had access to a clean source of lighting, solar powered lamps (49%).

HAP exposure was estimated based on self-reported household characteristics. The estimated personal exposure to fine particulate matter (PM_2.5_) was median 145 µg/m^3^ (IQR 79–270). There were 732 children (99.6%) with daily exposure exceeding 15 µg/m^3^, the limit recommended by WHO guidelines [17] and 484 (66%) exceeded 100 µg/m^3^, a level previously associated with higher risk of pneumonia [18].

**Fig 2:**
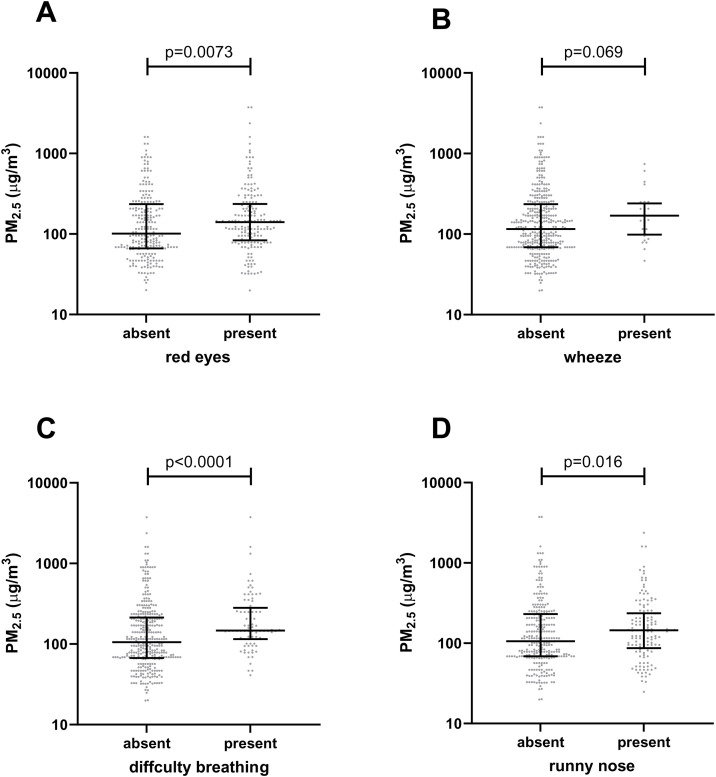
Association between parental report of chronic respiratory tract symptoms and estimated household air pollution (PM_2.5_). A. Red eyes; **B**. wheeze; **C**. difficulty breathing; and **D**. runny nose.

Parental report of symptoms related to smoke exposure included cough (57%), red eyes (43%), rhinorrhea (35%), difficulty breathing (21%) and wheeze (6.9%) (Table 2). Wood fuel was associated with 2.0-fold higher odds (95% CI 1.2–3.4) of red eyes relative to charcoal (p < 0.0001). Higher PM_2.5_ exposure was associated with a higher frequency of red eyes, difficulty breathing, and runny nose (Fig 2).

**Fig 3:**
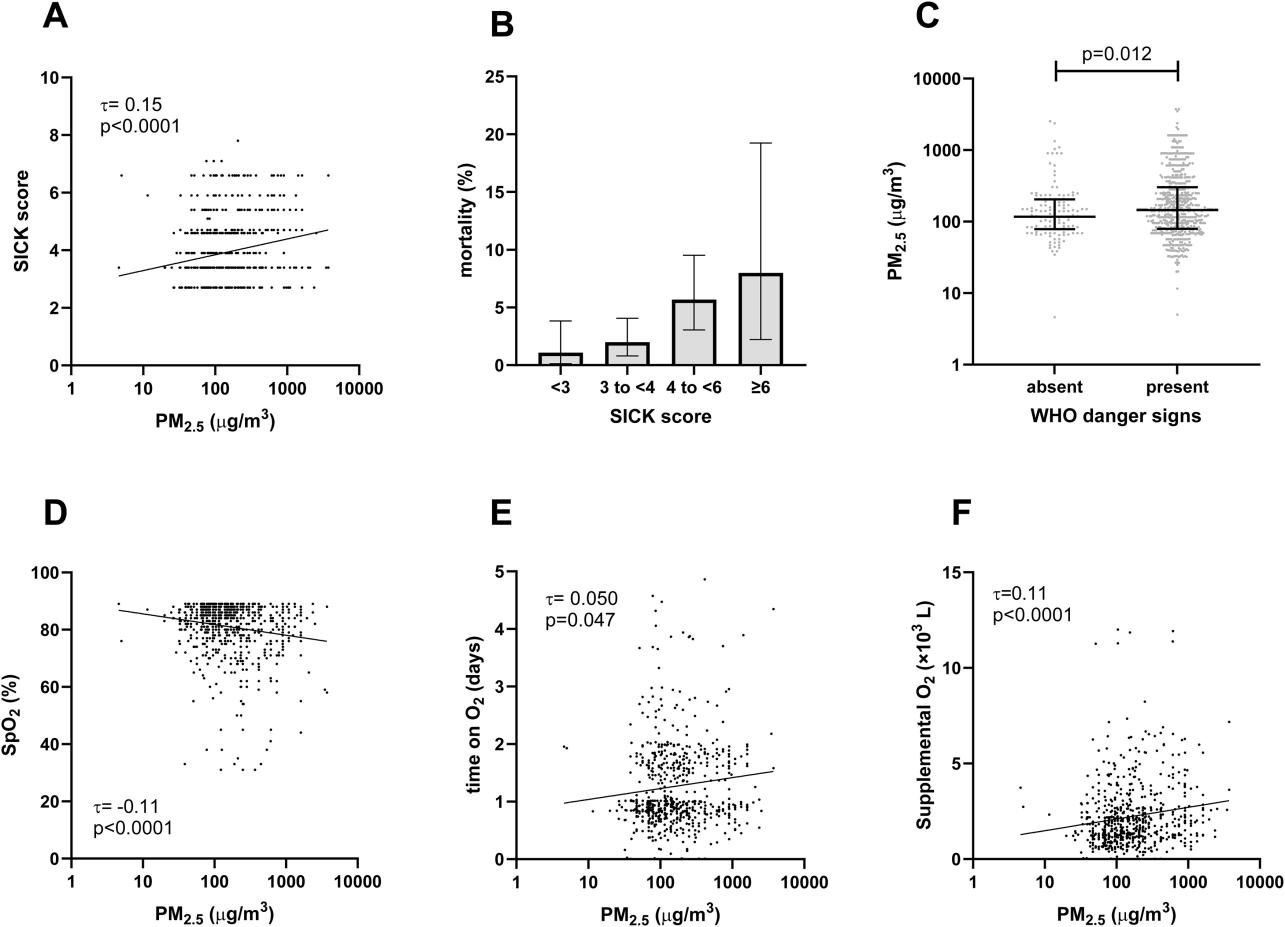
Association between disease severity and estimated daily average personal household air pollution exposure (PM_2.5_). A. There was a statistically significant correlation between the PM_2.5_ and composite severity score (Signs of Inflammation in Children that Kill, SICK, Kendall’s tau-B, τ = 0.15, p < 0.0001). B. Higher SICK score was associated with mortality. C. Higher PM_2.5_ was seen in children with WHO danger signs. **D-F**. The peripheral oxygen saturation (SpO_2_) was lower (D), the duration of oxygen therapy was longer (E), and the total volume of oxygen administered was higher (F) in patients exposed to higher levels of household air pollution.

The median composite disease severity score (SICK) was 3.9 (IQR 3.4 to 5.0). There was a statistically significant correlation between the PM_2.5_ and the SICK score (τ = 0.15, p < 0.0001, Fig 3A). This corresponds to a 0.52 point (95%CI 0.33–0.70, p < 0.0001) increase in the SICK score for every 10-fold increase in the PM_2.5_. In turn, higher SICK score was associated with higher risk of mortality (p = 0.0024, Fig 3B). Higher PM_2.5_ exposure was also associated with WHO danger signs (Fig 3C) and compromised oxygenation, as evidenced by lower SpO_2_ at admission, longer duration of oxygen therapy, and larger total volume of supplemental O2 administered (Fig 3D-F).

## Discussion

Here we show an association between HAP and disease severity in a country-wide cohort of Ugandan children under five years of age hospitalized with hypoxemic pneumonia. We found a statistically significant correlation between PM_2.5_ exposure and the SICK score (τ = 0.15, p < 0.0001), which, in turn, was linked to a higher risk of mortality. Our study confirms and extends previous findings from other LMICs. Previous studies have explored the association between HAP and childhood pneumonia, using a variety of study designs and methods to measure both exposure (HAP) and outcome (pneumonia incidence or severity) (Table 3). A systematic review and meta-analysis of published studies concluded that the odds of childhood pneumonia are approximately 1.8 times higher in households using unprocessed solid fuels than those using cleaner fuels (e.g., liquefied petroleum gas, kerosene, or electricity) [19]. Our study is noteworthy for its large sample size, its focus on hospitalized patients with severe pneumonia, and its validated method of estimating PM_2.5_.

**Table 3:** Association between household air pollution (HAP) and childhood pneumonia in previous studies.

First Author	Year	Country	Design	Number of Patients	Exposure (HAP)	Outcome	Magnitude of Association	Ref
**a.** **Studies that asked the caregiver about the type of household cooking fuel**								
Mishra	2006	India	Cross-sectional (survey)	29,768 children (0–35 months)	Questions on fuel type(s) mainly used for cooking: biofuels	ARI during the 2 weeks before interview (parental survey)	OR = 1.82 (95% CI 1.58–2.09)	[20]
Naz	2020	Pakistan	Cross-sectional (3 surveys)	28,919 children U5	Questions on fuel type(s) mainly used for cooking: polluting fuel	Childhood pneumonia based on maternal report of symptoms	OR = 1.25 (95% CI 1.05–1.35)	[21]
Sanbata	2014	Ethiopia	Cross-sectional	422 households, children U5	Questions on fuel type(s) mainly used for cooking: solid biomass fuel	ARI, based on maternal report of symptoms	aOR = 2.96 (95% CI 1.38–3.87)	[22]
Al-Sonboli	2006	Yemen	Case-control	601 children < 2 years old hospitalized	Questions on fuel type(s) mainly used for cooking: non-gas cooking fuels	ARI with hypoxemia (SpO_2_ < 88%) and RSV (RT-PCR)	OR = 10.3 (95% CI 2.2–48)	[23]
Islam	2022	Bangladesh	Cross-sectional (survey)	8321 children U5	Questions on fuel type(s) mainly used for cooking: solid fuels	ARI, based on caregiver report of symptoms	OR = 1.69 (95% CI 1.05–2.72)	[24]
Wichmann	2006	South Africa	Cross-sectional (survey)	4679 children U5	Questions on fuel type(s) mainly used for cooking: polluting fuels	ARI, based on maternal report of symptoms	OR = 1.27 (95% CI 1.05–1.55)	[25]
Bassani	2010	India	Case-control	616, 391 children U5	Fieldworker observation of fuel type used for cooking: solid fuel use	1. Non-fatal pneumonia 2. Child deaths in the previous year (household survey)	1. PR = 1.54 (boys) PR = 1.94 (girls) 2. PR = 1.30 (boys) PR = 1.33 (girls)	[26]
Dhimal	2010	Nepal	Cross-sectional	41 313 children U5	Questions on fuel type(s) mainly used for cooking: solid biomass fuel	ARI and pneumonia, based on caregiver recall and treatment records.	AF = 0.496	[27]
Mahalanabis	2002	India	Case-control	262 children aged 2–25 months	Questions on fuel type(s) mainly used for cooking: solid fuel	Physician-diagnosed pneumonia	OR = 3.97 (95% CI 2.00–7.88)	[28]
Budhathoki	2020	Nepal	Cross-sectional (3 surveys)	15, 372 children U5	Questions on fuel type(s) mainly used for cooking: polluting fuels	Childhood pneumonia, based on symptoms reported by parents	aRR = 1.98 (95% CI 1.01–3.92)	[29]
Bhat	2008	India	Case-control	214 children U5	Questions on fuel type(s) mainly used for cooking: fuel other than LPG	Clinically identified ALRI using WHO case definitions	OR = 4.63 (95% CI 1.67–12.97)	[30]
Jeena	2003	South Africa	Prospective observational	114 children aged 3–24 months	Questions on fuel type(s) mainly used for cooking: polluting fuel	RSV-confirmed bronchiolitis hospital admissions	Higher admission risk with noxious fuels (p = 0.05).	[31]
Sharma	1998	India	Prospective cohort	642 infants	Questions on fuel type(s) mainly used for cooking: wood or kerosene	Fieldworker-observed ALRI episodes during twice-weekly house visits	Kerosene: 31.7% ALRI Wood: 19.0% ALRI	[32]
**b.** **Studies that asked the caregiver about proximity to cooking fire**								
Armstrong	1991	The Gambia	Prospective cohort	~500 children U5	Questions on whether the child is carried by the mother during cooking	ALRI episodes identified via weekly field visits and chest X-rays	OR = 1.9 (95% CI 1.0–3.9)	[33]
De Francisco	1993	The Gambia	Case-control	129 children < 2 years old	Questions on whether the child is carried by the mother during cooking	ALRI deaths identified via verbal autopsy	aOR = 5.2 (95% CI 1.7–16)	[34]
O’Dempsey	1996	The Gambia	Case-control	239 children U5	Questions on whether the child is carried by the mother during cooking	Physician and microbiologic diagnosis of pneumococcal	OR = 2.55 (95% CI 0.98–6.65)	[35]
Howie	2016	The Gambia	Case-control	1581 children aged 2–59 months	Questions on whether the child is carried by the mother during cooking	Severe vs. non-severe pneumonia, based on presenting symptoms	aOR = 1.7 (95% CI 1.0–3.0)	[36]
Weber	1999	The Gambia	Case- control	641 children U5	Questions on whether the mother cooks at least once daily	Physician-diagnosed ALRI due to RSV	aOR = 0.31 (95% CI 0.14–0.70)	[37]
Pandey	1989	Nepal	Prospective observational	Eligible children < 2 years old	Questions on time spent near the fireplace per day >2 hrs/day near fireplace	Fieldworker-observed ARI episodes, graded by severity (Grades I-IV)	linked to higher ARI risk	[38]
Karki	2014	Nepal	Case-control	200 children U5	Questions regarding household use of a smoky cookstove located indoors	Physician-diagnosed pneumonia in hospitalized children	OR = 3.76 (95% CI 1.20–11.82)	[39]
PrayGod	2016	Tanzania	Case-control	117 children (2–59 months)	Questions on location of kitchen: indoor cooking	Physician-diagnosed severe or very severe pneumonia	OR = 5.5 (CI 1.4–22.1)	[40]
**c.** **Studies that measured HAP directly**								
Robin	1996	United States of America	Case-control	90 children aged 1–24 months	Domestic PM_10_ levels ≥ 65 μg/m^3^ (air-sampling pump)	Hospitalization for ALRI before interview (parental survey)	OR = 7.0 (95% CI 0.9–56.9)	[41]
Ezzati	2001	Kenya	Prospective cohort	94 children aged 0–4 years	Domestic PM_10_ levels >3500 µg/m³ (personal and stationary monitoring devices)	ARI, based on fieldworker observation of symptoms	OR = 6.73 (95% CI 3.75–12.06)	[42]
Kilabuko	2007	Tanzania	Cross-sectional	100 households	Domestic PM_10_, CO, and NO_2_ levels measured from biofuel cooking smoke	ARI diagnosed based on questionnaire answered by the household’s cook	OR = 5.5 (95% CI 3.6–8.5)	[43]
Gurley	2013	Bangladesh	Prospective cohort	257 children (followed 0–24 months)	Domestic PM_2.5_ levels >100 µg/m^3^ (PM air monitor)	Physician-diagnosed ALRI	aIRR = 1.07 (95% CI 1.01-1.14) per hour	[18]
**d.** **Study on smoking exposure**								
Shah	1994	India	Case-control	400 children U5	Questions on behavioral habits: smoking in household	Physician-diagnosed severe ARI	OR = 1.15 (95% CI 0.72–1.84)	[44]
**e.** **Randomized controlled trials in which the intervention aimed to reduce HAP**								
Smith	2011	Guatemala	RCT	534 households	Intervention to reduce HAP: woodstove with chimney	Physician-diagnosed pneumonia	RaR = 0.84 (95% CI 0.63–1.13)	[45]
Kinney	2021	Ghana	RCT	1141 infants	Intervention: clean cookstoves	Physician-diagnosed pneumonia and severe pneumonia	RR = 1.06 (95% CI 0.99–1,13)	[46]

ARI; acute respiratory infection; ALRI; acute lower respiratory infection; aRR, adjusted relative risk; OR, odds ratio; U5, under five years of age; PR, prevalence ratio; LPG, liquid petroleum gas; AF, attributable fraction; RaR, rate ratio; aIRR, adjusted incidence rate ratio; RCT, randomized controlled trial.

We estimated the median daily personal exposure to HAP (PM_2.5_) to be 145 µg/m^3^. This is higher than a recent multicentre study (e.g., median household exposure 48 µg/m^3^ in Blantyre, Malawi) [47] but similar to a 2013 study from Bangladesh, in which household PM_2.5_ concentrations exceeded 100 µg/m3 for a median of 5.3 hours per day [18]. We utilized a published method of estimating PM_2.5_ levels based on self-reported data, such as cooking fuel and kitchen type [9]. While many previous studies have similarly employed questionnaires asking about fuel use [18,20–25,27–30,32,40,41], whether the child is carried on the mother’s back during cooking [33–37], or general smoke exposure [31,38,39], others have attempted direct measurements of HAP by quantifying specific air pollutants, including CO [43,45,46,48], PM_2.5_ [18,22], NO_x_ [43], and PM_10_ [41,42,48,49]. Measurement methods included PM_2.5_ air samplers [18,22], PM_10_ air samplers [41,42,48,49], Lascar monitors [46], LD-3K fine dust monitors [43], and NO_2_ badges [43]. These approaches provide direct and objective measurements but are often complex, costly, and can be influenced by spatial and temporal variability in pollutant levels within and outside the household. While our study lacks direct HAP measurements and instead relies on self-reported survey data, it overcomes the limitations of simpler dichotomous exposure questionnaires (e.g., biofuels for cooking) by utilizing a validated log-linear model to estimate PM_2.5_ exposure [9]. Thus, our exposure measurement was quantitative yet practical. This may be a pragmatic, feasible, reproducible model for future studies of HAP in low-resource settings.

We examined the severity of pneumonia among hospitalized children as our primary outcome of interest, using the composite clinical severity score, SICK [11]. Past studies have used alternative outcomes relevant to childhood pneumonia, including caregiver-reported symptoms such as rapid or difficult breathing and cough [18,20–22,24,25,29,34,45,50], fieldworker observations of ARI symptoms during home visits [18,32,33,38,42,46,50], and diagnoses made by physicians [18,28,30,35,36,39,40,44,45,50], though standardized protocols were not always reported. Other studies have used more objective definitions of pneumonia, including radiological findings [33,50], assessments of oxygen saturation [23,50], or laboratory tests for respiratory syncytial virus (RSV) [23,31,37] or pneumococcus [35]. Some studies were community-based, in which most pneumonia episodes were likely mild [21,22,27,29,33]. Fewer studies examined hospitalized patients [28,31,36,37,39,41,44], severe pneumonia episodes [36,40,44,46], and fatal cases [26,34,50]. Our study is noteworthy for its inclusion of hospitalized, hypoxemic patients at high risk of mortality. Pneumonia severity was measured objectively, quantitatively, and in a standardized manner using the validated SICK clinical score. While past studies have demonstrated the association between HAP and incident pneumonia, our study links HAP to pneumonia severity in a cohort at high risk of mortality.

Supporting the deleterious effects of HAP, symptoms of airway and mucous membrane inflammation were common in our study. These observations are consistent with previous findings, where children exposed to biofuel smoke exhibited similar symptoms [51,52]. Such effects are biologically plausible because air pollutants can cause inflammation of the airways and alveoli, increasing the severity of respiratory infections [53]. Moreover, children under five may be more susceptible to the effects of HAP than adults for several reasons: shorter stature with higher exposure to dense smoke; immature immune defense mechanisms [53]; narrower airways, which result in greater proportional airway obstruction; and a larger lung surface area per kilogram of body weight, along with a higher oxygen consumption rate, leading to increased inhalation of polluted air [54]. We specifically found that higher PM_2.5_ concentrations are associated with an increased prevalence of rhinorrhea, difficulty breathing, and red eyes. Consistent with these findings, others observed a 39% increase in hourly cough rate in response to a 10-fold rise in hourly PM_2.5_ concentrations [55].

Our study provided rich data on household cooking practices in rural Uganda, which may influence childhood exposure to HAP. Firewood remains the predominant cooking fuel, used by 84% of households. This reliance on firewood combustion may contribute to higher PM_2.5_ exposure levels due to greater emissions compared to charcoal. For instance, charcoal emits lower PM_2.5_ levels, averaging 0.21 ± 0.06 ppm [56], and has a lower PM_2.5_ to PM_10_ ratio than firewood [57]. Yet, despite being less polluting and offering shorter cooking times due to its higher burning efficiency (28% as opposed to 17%), charcoal adoption remains limited [58]. Its higher cost makes it less accessible than firewood, which is often freely available in rural areas. Consistent with our findings, firewood is often left burning long after cooking is completed, as seen in Kenyan households where cooking fires were reported to burn for 5–12 hours daily, further exacerbating PM_2.5_ exposure [59]. Since firewood does not incur a monetary cost, families may allow it to continue burning even after cooking is complete, which may explain the longer cooking durations compared to charcoal. However, cooking with charcoal is far from an ideal alternative, and innovations in cleaner cooking technologies suitable for low-income settings are actively being explored. Households using cleaner fuel alternatives such as liquefied petroleum gas [60], ethanol cookstoves [61], and solar-powered cookstoves [62] represent important advances toward reducing HAP exposure. In parallel, chimney-equipped biomass stoves have shown potential to reduce HAP, with previous findings demonstrating an approximately 90% reduction in 48-hour kitchen CO concentrations compared to open wood fires [45]. Hence, these alternative cooking methods represent promising interventions for reducing pneumonia-related childhood morbidity and mortality, especially in rural communities. In addition, cost-effective behavioural change interventions (typically delivered through community counseling), such as promoting outdoor cooking, increasing ventilation while cooking indoors, and minimizing children’s exposure to traditional indoor cooking fires, have also been effective in reducing household PM_10_ and CO concentrations [63].

Our study has several limitations. This was a secondary analysis of data originally collected for a cluster randomized controlled trial [8]. We retrospectively assessed the exposure through parental reports of household characteristics and cooking practices. Thus, it may be subject to recall bias, limiting our ability to make causal inferences. Furthermore, we used modeled PM_2.5_ exposure estimates based on household variables and calculated an estimated PM_2.5_ exposure. While this estimate has a moderate correlation with measured values of PM_2.5_, it remains a superior alternative to simple dichotomous fuel-type questionnaires, which are the standard in much of the existing literature [9]. This may have reduced the precision of the exposure assessment. Although our sample size was larger than many past publications, a study with more fatal cases would be necessary to show a direct association between HAP and mortality. Nonetheless, we demonstrated a plausible relationship between HAP and disease severity, and between disease severity and death. Our multi-center study included 20 Ugandan health facilities, which are likely representative of the country’s public hospitals; however, the population was uniformly rural, low-income, and highly exposed to HAP through firewood cooking smoke. Results should therefore be extrapolated with caution to other countries, higher-income settings, and areas with lower use of cooking biofuels.

In summary, HAP appears to be a modifiable risk factor for severe childhood pneumonia, a leading cause of mortality among children under five globally. Our findings support initiatives aimed at reducing HAP as a strategy toward improved global child survival [64].

## Supporting information

S1 Formula Equation to predict household concentrations of particulate matter <2.5 µm in size (PM_2.5_). (DOCX)

S2 Formula Equation to calculate the sample size for a Pearson correlation coefficient. (DOCX)

## References

[1] Liu L, Oza S, Hogan D, Chu Y, Perin J, Zhu J, et al. Global, regional, and national causes of under-5 mortality in 2000-15: an updated systematic analysis with implications for the Sustainable Development Goals. Lancet. 2016;388(10063):3027–35. doi: 10.1016/S0140-6736(16)31593-8 27839855 PMC5161777

[2] Wardlaw TM, Johansson EW, Hodge M, World Health Organization, United Nations Children’s Fund. Pneumonia: the forgotten killer of children. Geneva: World Health Organization. 2006.

[3] Ezzati M, Kammen D. Indoor air pollution from biomass combustion and acute respiratory infections in Kenya: an exposure-response study. Lancet. 2001;358(9282):619–24. doi: 10.1016/s0140-6736(01)05777-4 11530148

[4] Gall ET, Carter EM, Earnest CM, Stephens B. Indoor air pollution in developing countries: research and implementation needs for improvements in global public health. Am J Public Health. 2013;103(4):e67-72. doi: 10.2105/AJPH.2012.300955 23409891 PMC3673244

[5] Kurmi OP, Lam KBH, Ayres JG. Indoor air pollution and the lung in low- and medium-income countries. Eur Respir J. 2012;40(1):239–54. doi: 10.1183/09031936.00190211 22362845

[6] Ketzel M, Omstedt G, Johansson C, Düring I, Pohjola M, Oettl D. Estimation and validation of PM2.5/PM10 exhaust and non-exhaust emission factors for practical street pollution modelling. Atmos Environ. 2007;41(40):9370–85. doi: 10.1016/j.atmosenv.2007.09.005

[7] Raju S, Siddharthan T, McCormack MC. Indoor air pollution and respiratory health. Clin Chest Med. 2020;41(4):825–43. doi: 10.1016/j.ccm.2020.08.014 33153698 PMC7665158

[8] Conradi N, Opoka RO, Mian Q, Conroy AL, Hermann LL, Charles O, et al. Solar-powered O2 delivery for the treatment of children with hypoxaemia in Uganda: a stepped-wedge, cluster randomised controlled trial. Lancet. 2024;403(10428):756–65. doi: 10.1016/S0140-6736(23)02502-3 38367643

[9] Balakrishnan K, Ghosh S, Ganguli B, Sambandam S, Bruce N, Barnes DF, et al. State and national household concentrations of PM2.5 from solid cookfuel use: results from measurements and modeling in India for estimation of the global burden of disease. Environ Health. 2013;12(1):77. doi: 10.1186/1476-069X-12-77 24020494 PMC3851863

[10] Smith KR, Bruce N, Balakrishnan K, Adair-Rohani H, Balmes J, Chafe Z, et al. Millions dead: how do we know and what does it mean? Methods used in the comparative risk assessment of household air pollution. Annu Rev Public Health. 2014;35:185–206. doi: 10.1146/annurev-publhealth-032013-182356 24641558

[11] Gupta MA, Chakrabarty A, Halstead R, Sahni M, Rangasami J, Puliyel A, et al. Validation of “Signs of Inflammation in Children that Kill” (SICK) score for immediate non-invasive assessment of severity of illness. Ital J Pediatr. 2010;36:35. doi: 10.1186/1824-7288-36-35 20420670 PMC2873401

[12] Bhal S, Tygai V, Kumar N, Sreenivas V, Puliyel JM. Signs of inflammation in children that can kill (SICK score): preliminary prospective validation of a new non-invasive measure of severity-of-illness. J Postgrad Med. 2006;52(2):102–5. 16679672

[13] Bulus M. pwrss: Statistical Power and Sample Size Calculation Tools. 0.3.1 ed: R package; 2023.

[14] Alkire S, Foster J. Counting and multidimensional poverty measurement. Journal of Public Economics. 2011;95(7–8):476–87. doi: 10.1016/j.jpubeco.2010.11.006

[15] WHO. The WHO Child Growth Standards. http://www.who.int/childgrowth/standards/en/ Accessed 15 December 2022. 2006.

[16] Fleming S, Thompson M, Stevens R, Heneghan C, Plüddemann A, Maconochie I, et al. Normal ranges of heart rate and respiratory rate in children from birth to 18 years of age: a systematic review of observational studies. Lancet. 2011;377(9770):1011–8. doi: 10.1016/S0140-6736(10)62226-X 21411136 PMC3789232

[17] Lim S, Bassey E, Bos B, Makacha L, Varaden D, Arku RE, et al. Comparing human exposure to fine particulate matter in low and high-income countries: A systematic review of studies measuring personal PM2.5 exposure. Sci Total Environ. 2022;833:155207. doi: 10.1016/j.scitotenv.2022.155207 35421472 PMC7615091

[18] Gurley ES, Homaira N, Salje H, Ram PK, Haque R, Petri W, et al. Indoor exposure to particulate matter and the incidence of acute lower respiratory infections among children: a birth cohort study in urban Bangladesh. Indoor Air. 2013;23(5):379–86. doi: 10.1111/ina.12038 23906055 PMC3773273

[19] Dherani M, Pope D, Mascarenhas M, Smith KR, Weber M, Bruce N. Indoor air pollution from unprocessed solid fuel use and pneumonia risk in children aged under five years: a systematic review and meta-analysis. Bull World Health Organ. 2008;86(5):390-398C. doi: 10.2471/blt.07.044529 18545742 PMC2647443

[20] Mishra V, Smith KR, Retherford RD. Effects of Cooking Smoke and Environmental Tobacco Smoke on Acute Respiratory Infections in Young Indian Children. Popul Environ. 2005;26(5):375–96. doi: 10.1007/s11111-005-0005-y

[21] Naz L, Ghimire U. Assessing the prevalence trend of childhood pneumonia associated with indoor air pollution in Pakistan. Environ Sci Pollut Res Int. 2020;27(35):44540–51. doi: 10.1007/s11356-020-10346-6 32770471

[22] Sanbata H, Asfaw A, Kumie A. Association of biomass fuel use with acute respiratory infections among under- five children in a slum urban of Addis Ababa, Ethiopia. BMC Public Health. 2014;14:1122. doi: 10.1186/1471-2458-14-1122 25358245 PMC4237768

[23] Al-Sonboli N, Hart CA, Al-Aghbari N, Al-Ansi A, Ashoor O, Cuevas LE. Human metapneumovirus and respiratory syncytial virus disease in children, Yemen. Emerg Infect Dis. 2006;12(9):1437–9. doi: 10.3201/eid1209.060207 17073098 PMC3294747

[24] Islam MA, Hasan MN, Ahammed T, Anjum A, Majumder A, Siddiqui MN-E-A, et al. Association of household fuel with acute respiratory infection (ARI) under-five years children in Bangladesh. Front Public Health. 2022;10:985445. doi: 10.3389/fpubh.2022.985445 36530721 PMC9752885

[25] Wichmann J, Voyi KVV. Impact of cooking and heating fuel use on acute respiratory health of preschool children in South Africa. Southern African Journal of Epidemiology and Infection. 2006;21(2):48–54. doi: 10.1080/10158782.2006.11441264

[26] Bassani DG, Jha P, Dhingra N, Kumar R. Child mortality from solid-fuel use in India: a nationally-representative case-control study. BMC Public Health. 2010;10:491. doi: 10.1186/1471-2458-10-491 20716354 PMC2931474

[27] Dhimal M, Dhakal P, Shrestha N, Baral K, Maskey M. Environmental burden of acute respiratory infection and pneumonia due to indoor smoke in Dhading. J Nepal Health Res Counc. 2010;8(1):1–4. 21879004

[28] Mahalanabis D, Gupta S, Paul D, Gupta A, Lahiri M, Khaled MA. Risk factors for pneumonia in infants and young children and the role of solid fuel for cooking: a case-control study. Epidemiol Infect. 2002;129(1):65–71. doi: 10.1017/s0950268802006817 12211598 PMC2869876

[29] Budhathoki SS, Tinkari BS, Bhandari A, Dhimal M, Zhou H, Ghimire A, et al. The Association of Childhood Pneumonia with Household Air Pollution in Nepal: Evidence from Nepal Demographic Health Surveys. Matern Child Health J. 2020;24(Suppl 1):48–56. doi: 10.1007/s10995-020-02882-x 31981064 PMC7048702

[30] Bhat YR, Dhanya Y, Sanjay D, Manjunath N, Sithara R. Risk Factors for Acute Lower Respiratory Tract Infections in Under-five Children of Developing Country. International Journal of Infectious Diseases. 2008;12:e64–5. doi: 10.1016/j.ijid.2008.05.161

[31] Jeena PM, Ayannusi OE, Annamalai K, Naidoo P, Coovadia HM, Guldner P. Risk factors for admission and the role of respiratory syncytial virus-specific cytotoxic T-lymphocyte responses in children with acute bronchiolitis. S Afr Med J. 2003;93(4):291–4. 12806723

[32] Sharma S, Sethi GR, Rohtagi A, Chaudhary A, Shankar R, Bapna JS, et al. Indoor air quality and acute lower respiratory infection in Indian urban slums. Environ Health Perspect. 1998;106(5):291–7. doi: 10.1289/ehp.98106291 9560355 PMC1533083

[33] Armstrong JR, Campbell H. Indoor air pollution exposure and lower respiratory infections in young Gambian children. Int J Epidemiol. 1991;20(2):424–9. doi: 10.1093/ije/20.2.424 1917245

[34] de Francisco A, Morris J, Hall AJ, Armstrong Schellenberg JR, Greenwood BM. Risk factors for mortality from acute lower respiratory tract infections in young Gambian children. Int J Epidemiol. 1993;22(6):1174–82. doi: 10.1093/ije/22.6.1174 8144302

[35] O’Dempsey TJ, McArdle TF, Morris J, Lloyd-Evans N, Baldeh I, Laurence BE, et al. A study of risk factors for pneumococcal disease among children in a rural area of west Africa. Int J Epidemiol. 1996;25(4):885–93. doi: 10.1093/ije/25.4.885 8921471

[36] Howie SRC, Schellenberg J, Chimah O, Ideh RC, Ebruke BE, Oluwalana C, et al. Childhood pneumonia and crowding, bed-sharing and nutrition: a case-control study from The Gambia. Int J Tuberc Lung Dis. 2016;20(10):1405–15. doi: 10.5588/ijtld.15.0993 27725055 PMC5019143

[37] Weber MW, Milligan P, Hilton S, Lahai G, Whittle H, Mulholland EK, et al. Risk factors for severe respiratory syncytial virus infection leading to hospital admission in children in the Western Region of The Gambia. Int J Epidemiol. 1999;28(1):157–62. doi: 10.1093/ije/28.1.157 10195682

[38] Pandey MR, Neupane RP, Gautam A, Shrestha IB. Domestic smoke pollution and acute respiratory infections in a rural community of the hill region of Nepal. Environment International. 1989;15(1–6):337–40. doi: 10.1016/0160-4120(89)90046-9

[39] Karki S, Fitzpatrick AL, Shrestha S. Risk Factors for Pneumonia in Children under 5 Years in a Teaching Hospital in Nepal. Kathmandu Univ Med J (KUMJ). 2014;12(48):247–52. doi: 10.3126/kumj.v12i4.13729 26333578

[40] PrayGod G, Mukerebe C, Magawa R, Jeremiah K, Török ME. Indoor Air Pollution and Delayed Measles Vaccination Increase the Risk of Severe Pneumonia in Children: Results from a Case-Control Study in Mwanza, Tanzania. PLoS One. 2016;11(8):e0160804. doi: 10.1371/journal.pone.0160804 27508389 PMC4979871

[41] Robin LF, Less PS, Winget M, Steinhoff M, Moulton LH, Santosham M, et al. Wood-burning stoves and lower respiratory illnesses in Navajo children. Pediatr Infect Dis J. 1996;15(10):859–65. doi: 10.1097/00006454-199610000-00006 8895916

[42] Ezzati M, Kammen DM. Quantifying the effects of exposure to indoor air pollution from biomass combustion on acute respiratory infections in developing countries. Environ Health Perspect. 2001;109(5):481–8. doi: 10.1289/ehp.01109481 11401759 PMC1240307

[43] Kilabuko JH, Matsuki H, Nakai S. Air quality and acute respiratory illness in biomass fuel using homes in Bagamoyo, Tanzania. Int J Environ Res Public Health. 2007;4(1):39–44. doi: 10.3390/ijerph2007010007 17431314 PMC3719958

[44] Shah N, Ramankutty V, Premila PG, Sathy N. Risk factors for severe pneumonia in children in south Kerala: a hospital-based case-control study. J Trop Pediatr. 1994;40(4):201–6. doi: 10.1093/tropej/40.4.201 7932932

[45] Smith KR, McCracken JP, Weber MW, Hubbard A, Jenny A, Thompson LM, et al. Effect of reduction in household air pollution on childhood pneumonia in Guatemala (RESPIRE): a randomised controlled trial. Lancet. 2011;378(9804):1717–26. doi: 10.1016/S0140-6736(11)60921-5 22078686

[46] Kinney PL, Asante K-P, Lee AG, Ae-Ngibise KA, Burkart K, Boamah-Kaali E, et al. Prenatal and Postnatal Household Air Pollution Exposures and Pneumonia Risk: Evidence From the Ghana Randomized Air Pollution and Health Study. Chest. 2021;160(5):1634–44. doi: 10.1016/j.chest.2021.06.080 34298005 PMC8628168

[47] Lim S, Said B, Zurba L, Mosler G, Addo-Yobo E, Adeyeye OO, et al. Characterising sources of PM2·5 exposure for school children with asthma: a personal exposure study across six cities in sub-Saharan Africa. Lancet Child Adolesc Health. 2024;8(1):17–27. doi: 10.1016/S2352-4642(23)00261-4 38000380 PMC10716619

[48] Röllin HB, Mathee A, Bruce N, Levin J, von Schirnding YER. Comparison of indoor air quality in electrified and un-electrified dwellings in rural South African villages. Indoor Air. 2004;14(3):208–16. doi: 10.1111/j.1600-0668.2004.00238.x 15104789

[49] Dasgupta S, Wheeler D, Huq M, Khaliquzzaman M. Improving indoor air quality for poor families: a controlled experiment in Bangladesh. Indoor Air. 2009;19(1):22–32. doi: 10.1111/j.1600-0668.2008.00558.x 19191925

[50] Bruce N, Weber M, Arana B, Diaz A, Jenny A, Thompson L, et al. Pneumonia case-finding in the RESPIRE Guatemala indoor air pollution trial: standardizing methods for resource-poor settings. Bull World Health Organ. 2007;85(7):535–44. doi: 10.2471/blt.06.035832 17768502 PMC2636369

[51] Dida GO, Lutta PO, Abuom PO, Mestrovic T, Anyona DN. Factors predisposing women and children to indoor air pollution in rural villages, Western Kenya. Arch Public Health. 2022;80(1):46. doi: 10.1186/s13690-022-00791-9 35093174 PMC8801101

[52] Khalequzzaman M, Kamijima M, Sakai K, Chowdhury NA, Hamajima N, Nakajima T. Indoor air pollution and its impact on children under five years old in Bangladesh. Indoor Air. 2007;17(4):297–304. doi: 10.1111/j.1600-0668.2007.00477.x 17661926

[53] Smith KR, Samet JM, Romieu I, Bruce N. Indoor air pollution in developing countries and acute lower respiratory infections in children. Thorax. 2000;55(6):518–32. doi: 10.1136/thorax.55.6.518 10817802 PMC1745777

[54] Moya J, Bearer CF, Etzel RA. Children’s behavior and physiology and how it affects exposure to environmental contaminants. Pediatrics. 2004;113(4 Suppl):996–1006. 15060192

[55] Zimmer AJ, Tsang LY, Jolicoeur G, Tannir B, Batisse E, Pando C, et al. Incidence of cough from acute exposure to fine particulate matter (PM2.5) in Madagascar: A pilot study. PLOS Glob Public Health. 2024;4(7):e0003530. doi: 10.1371/journal.pgph.0003530 39058715 PMC11280240

[56] Oladosu KO, Babalola SA, Kareem MW, Ajimotokan HA, Kolawole MY, Issa WA, et al. Optimization of fuel briquette made from bi-composite biomass for domestic heating applications. Scientific African. 2023;21:e01824. doi: 10.1016/j.sciaf.2023.e01824

[57] Lea-Langton AR, Spracklen DV, Arnold SR, Conibear LA, Chan J, Mitchell EJS, et al. PAH emissions from an African cookstove. Journal of the Energy Institute. 2019;92(3):587–93. doi: 10.1016/j.joei.2018.03.014

[58] Smith JU, Fischer A, Hallett PD, Homans HY, Smith P, Abdul-Salam Y, et al. Sustainable use of organic resources for bioenergy, food and water provision in rural Sub-Saharan Africa. Renewable and Sustainable Energy Reviews. 2015;50:903–17. doi: 10.1016/j.rser.2015.04.071

[59] Boleij JS, Brunekreef B. Domestic pollution as a factor causing respiratory health effects. Chest. 1989;96(3 Suppl):368S-372S. doi: 10.1378/chest.96.3_supplement.368s 2670478

[60] Shupler M, Tawiah T, Nix E, Baame M, Lorenzetti F, Betang E, et al. Household concentrations and female and child exposures to air pollution in peri-urban sub-Saharan Africa: measurements from the CLEAN-Air(Africa) study. Lancet Planet Health. 2024;8(2):e95–107. doi: 10.1016/S2542-5196(23)00272-3 38331535 PMC10864747

[61] Benka-Coker ML, Tadele W, Milano A, Getaneh D, Stokes H. A case study of the ethanol CleanCook stove intervention and potential scale-up in Ethiopia. Energy Sustain Dev. 2018;46:53–64. doi: 10.1016/j.esd.2018.06.009 30918423 PMC6433382

[62] Kashyap SR, Pramanik S, Ravikrishna RV. A review of solar, electric and hybrid cookstoves. Renewable and Sustainable Energy Reviews. 2023;188:113787. doi: 10.1016/j.rser.2023.113787

[63] Barnes B, Mathee A, Thomas E. The impact of health behaviour change intervention on indoor air pollution indicators in the rural North West Province, South Africa. J energy South Afr. 2011;22(3):35–44. doi: 10.17159/2413-3051/2011/v22i3a3220

[64] Howie SRC, Murdoch DR. Global childhood pneumonia: the good news, the bad news, and the way ahead. Lancet Glob Health. 2019;7(1):e4–5. doi: 10.1016/S2214-109X(18)30446-7 30497987

