# Supplementary material for: Household air pollution is associated with disease severity in Ugandan children hospitalized with hypoxemic pneumonia
Source: PLoS One. 2026 May 12;21(5):e0348277. doi: 10.1371/journal.pone.0348277 (PMC13166932; doi:10.1371/journal.pone.0348277)
Supplement: S1 Formula — (DOCX) [file pone.0348277.s001.docx]

**Supporting information**

**S1 Formula. Equation to predict household concentrations of particulate matter <2.5 µm in size (PM_2.5_)**

The following log-linear regression model [1] was used to estimate the PM_2.5_ in the kitchen area from household characteristics:

$$E \{log({PM}_{2.5})\}=\beta_{0}+$$

$\beta_{F1}I \left( Fuel=Kerosene \right)+\beta_{F3}I \left( Fuel=Wood or charcoal \right)+$

$$\beta_{K1}I \left( Kit=SOK \right)+\beta_{K2}I \left( Kit=IDK \right)+$$

$$\beta_{V1}I \left( Vent=Moderate \right)+\beta_{V2}I \left( Vent=Poor \right)+$$

$$\beta_{CH}I \left( Cooking hours \right)$$

where $I \left( X=L \right)$ = 1, if the categorical variable *X* assumes the level ‘L’, else 0.

Reference categories were: Liquefied petroleum gas (LPG) for fuel; outdoor kitchen (ODK) for kitchen type/location; and “good” for ventilation.

| **Parameter** ^a^ | **Coefficient** | **Value** |
| --- | --- | --- |
| Intercept | $\beta_{0}$ | -1.653 |
| Fuel ^b^: kerosene vs. LPG | $\beta_{F1}$ | 0.194 |
| Fuel ^b^: wood or charcoal vs. LPG | $\beta_{F3}$ | 0.969 |
| Kitchen: SOK vs. ODK | $\beta_{K1}$ | -0.389 |
| Kitchen: IDK vs. ODK | $\beta_{K2}$ | -0.594 |
| Ventilation: moderate vs. good | $\beta_{V1}$ | -0.082 |
| Ventilation: poor vs. good | $\beta_{V2}$ | -0.391 |
| Cooking hours | $\beta_{CH}$ | 0.084 |

ODK, outdoor kitchen; IDK, indoor kitchen; SOK, separate outdoor kitchen; LPG, Liquified petroleum gas

^a^ The region of India was included in the Balakrishnan model [1], but not here.

^b^ Dung was not used as a fuel source by any study participants; therefore, its coefficient from the original Balakrishnan model [1] is not included here.

The daily average personal exposure of a young child was calculated from the kitchen area concentration as follows [2]:

$${PM}_{2.5}\left( daily average \right)= 0.628\times{PM}_{2.5}(kitchen)$$

**References**

1. Balakrishnan K, Ghosh S, Ganguli B, Sambandam S, Bruce N, Barnes DF, et al. State and national household concentrations of PM2.5 from solid cookfuel use: results from measurements and modeling in India for estimation of the global burden of disease. Environ Health. 2013;12(1):77. Epub 20130911. doi: 10.1186/1476-069X-12-77. PubMed PMID: 24020494; PubMed Central PMCID: PMCPMC3851863.

2. Smith KR, Bruce N, Balakrishnan K, Adair-Rohani H, Balmes J, Chafe Z, et al. Millions dead: how do we know and what does it mean? Methods used in the comparative risk assessment of household air pollution. Annu Rev Public Health. 2014;35:185-206. doi: 10.1146/annurev-publhealth-032013-182356. PubMed PMID: 24641558.
