# Supplementary material for: Household air pollution is associated with disease severity in Ugandan children hospitalized with hypoxemic pneumonia
Source: PLoS One. 2026 May 12;21(5):e0348277. doi: 10.1371/journal.pone.0348277 (PMC13166932; doi:10.1371/journal.pone.0348277)
Supplement: S2 Formula — (DOCX) [file pone.0348277.s002.docx]

**Supporting information**

**S2 Formula. Equation to calculate the sample size for a Pearson correlation coefficient**

$$H_{0}:\rho\leq0$$

$$H_{a}:\rho>0$$

$n=\left( \frac{Z_{1-\alpha}+Z_{1-\beta}}{z_{r_{a}}} \right)^{2}$+3

$$z_{r_{a}}=0.5\times ln\left( \frac{1+r_{a}}{1-r_{a}} \right)$$

Where:

$\rho$ is the population correlation coefficient

$r_{a}$ is the expected correlation

$n$ is the desired sample size

$Z$ represents the critical values for the alpha level and power

$Z_{1-\alpha}\approx1.64$ at $\alpha=0.05$ (one-sided)

$Z_{1-\beta}\approx0.84$ at $\beta=0.2$
