# Supplementary material for: Household air pollution is associated with disease severity in Ugandan children hospitalized with hypoxemic pneumonia
Source: PLoS One. 2026 May 12;21(5):e0348277. doi: 10.1371/journal.pone.0348277 (PMC13166932; doi:10.1371/journal.pone.0348277)
Supplement: Attachment — Submitted filename: Review - HOUSEHOLD AIR POLLUTION .docx [file pone.0348277.s003.docx]

**REVIEW**

**of the article:**

**«HOUSEHOLD AIR POLLUTION IS ASSOCIATED WITH DISEASE SEVERITY IN UGANDAN CHILDREN HOSPITALIZED WITH HYPOXEMIC PNEUMONIA»**

The study examines the incidence of pneumonia among children in Uganda in connection with air pollution from domestic stoves. The topic of the research is relevant because children's bodies are most sensitive to harmful factors, and most pollutants enter the body through inhalation. In addition, despite its low level of industrialisation and, accordingly, low emissions from industrial enterprises, Uganda is characterised by elevated levels of PM2.5 air pollution (according to data from open sources https://waqi.info/). Therefore, identifying sources of pollution and determining the consequences for public health is an important and rather difficult task in the absence of an effective air monitoring system in the studied area.

To determine the concentrations of pollutants and their exposure to assess the health risks to children, the study uses computational and statistical assessment methods, as well as questionnaires on living conditions, including the use of domestic stoves during food preparation. This approach seems acceptable and compromise option in the absence of data from a monitoring system based on instrumental control of atmospheric pollution parameters. A sample of 735 children who were hospitalised with pneumonia is sufficient for statistical analysis of the causal relationships between exposure and morbidity based on the methodology used by the authors. The authors' idea to exclude children with specific diseases such as heart defects, ischaemic encephalopathy, malaria and others from the study group is very appropriate. The authors used specialised software, GraphPad Prism, for statistical analysis and data visualisation.

The research revealed a close link between indoor air pollution caused using organic fuel stoves and the severity of childhood pneumonia, mortality, etc. A comparative analysis of other researchers who measured atmospheric air concentrations in homes using both gas analysers and calculation methods is also presented. Thus, the high incidence of respiratory diseases among children in Uganda may be due to purely unsatisfactory living conditions. The studies conducted are of practical importance and social impact, as they provide a scientific basis for the search for safer ways of cooking at home.

The work does not contain personal data of respondents (parents) and hospitalised children with pneumonia, and the studies do not violate ethical standards and can be made public.

While reviewing the materials of the study, several questions and minor comments arose:

1. The study states that the average daily concentration of PM 2.5 is 145 μg per cubic metre. This is a rather dangerous concentration, as it is 10 times higher than the WHO standard. This is an important value for research, as it determines the prediction of risks to children's health. Therefore, it was necessary to clarify how this value was measured or calculated and to provide the calculation method or source for such a method.

2. The study should have listed the cities or towns in Uganda where the research was conducted. This does not violate the personal data of respondents but will allow for future analysis of data from the air pollution monitoring system on the streets of the studied areas to confirm that the health hazard to children comes specifically from the use of domestic stoves indoors.

3. It is known that stove heating and cooking are still used in many rural homes without centralised gas supply, including in Eastern European countries. However, carbon monoxide, which is produced by the incomplete combustion of organic fuel, is traditionally considered the main threat to residents there. In this regard, it was also worth describing the typical models of stoves used in Ugandan households.

The comments received are minor and are purely advisory in nature.

Conclusion. This publication contains all the necessary components and characteristics required for scientific work, namely, it raises a topical hygiene issue, provides an overview of retrospective studies on the selected topic and discusses the results obtained, and offers research novelty and practical recommendations. The results of the studies are of interest to researchers and specialists in various fields, in particular ecology and hygiene of populated cities, analysis of medical and statistical data, development of atmospheric air monitoring systems, etc. The work is formatted in accordance with the standards, supporting research materials are included in the appendices, and the list of references corresponds to the sources. I believe that this publication, in terms of the nature, content and scope of the research conducted, meets the requirements of the PLOS One journal and can be published.

Buchavyi Yurii, PhD (biology)

Associate Professor,

The Department of Ecology and

Technologies of Environmental Protection,

Dnipro University of Technology
